# OpenSAFELY: Effectiveness of COVID-19 Vaccination in Children and Adolescents

**DOI:** 10.1097/EDE.0000000000001908

**Published:** 2025-09-23

**Authors:** Colm D. Andrews, Edward P. K. Parker, Elsie Horne, Venexia Walker, Tom Palmer, Andrea L. Schaffer, Amelia C. A. Green, Helen J. Curtis, Alex J. Walker, Lucy Bridges, Christopher Wood, Victoria Speed, Christopher Bates, Jonathan Cockburn, John Parry, Amir Mehrkar, Brian MacKenna, Sebastian C. J. Bacon, Ben Goldacre, Miguel A. Hernan, Jonathan A. C. Sterne, William J. Hulme, on Behalf of the OpenSAFELY Collaborative

**Affiliations:** From the a Nuffield Department of Primary Care Health Sciences, Bennett Institute for Applied Data Science, University of Oxford, Oxford, United Kingdom; b London School of Hygiene and Tropical Medicine, London, United Kingdom; c Population Health Sciences, University of Bristol, Bristol, United Kingdom; d TPP, TPP House, Leeds, United Kingdom; e Departments of Epidemiology and Biostatistics, Harvard T.H. Chan School of Public Health, Boston, MA.

**Keywords:** Coronavirus, COVID, Epidemiology, Pandemics, Pediatrics, Vaccines

## Abstract

**Background:**

We assessed the safety and effectiveness of the first- and second-dose BNT162b2 COVID-19 vaccination, offered as part of the national COVID-19 vaccine roll-out from September 2021, in children and adolescents in England.

**Methods:**

Our observational study using OpenSAFELY-TPP, included adolescents aged 12–15 years and children aged 5–11 years. It compared individuals receiving (1) the first vaccination to unvaccinated controls and (2) the second vaccination to single-vaccinated controls. We matched vaccinated individuals with controls on age, sex, and other important characteristics. Outcomes were positive SARS-CoV-2 test (adolescents only), COVID-19 accident and emergency (A&E) attendance, COVID-19 hospitalization, COVID-19 critical care admission, and COVID-19 death; with safety outcomes, A&E attendance, unplanned hospitalization, pericarditis, and myocarditis.

**Results:**

Among 820,926 previously unvaccinated adolescents, 20-week incidence rate ratios (IRRs) comparing vaccination with no vaccination were 0.74 for positive SARS-CoV-2 test, 0.60 for COVID-19 A&E attendance, and 0.58 for COVID-19 hospitalization. Among 441,858 adolescents who had received the first vaccination, IRRs comparing second dose with single-vaccination were 0.67 for positive SARS-CoV-2 test, 1.00 for COVID-19 A&E attendance, and 0.60 for COVID-19 hospitalization. In both children groups, COVID-19-related outcomes were too rare to allow IRRs to be estimated precisely. Across all analyses, there were no COVID-19-related deaths, and fewer than seven COVID-19-related critical care admissions. Myocarditis and pericarditis were documented only in the vaccinated groups, with rates of 27 and 10 cases/million after the first and second doses, respectively.

**Conclusions:**

BNT162b2 vaccination in adolescents reduced COVID-19 A&E attendance and hospitalization, although these outcomes were rare. Protection against positive SARS-CoV-2 tests was transient.

The United Kingdom extended its COVID-19 vaccination program to adolescents aged 12–15 years on 20 September 2021, with an adult 30 µg dose of Pfizer-BioNTech (BNT162b2) vaccine licensed for use.^1^ Children aged 5–11 years were eligible from 4 April 2022, using a 10 µg dose.^2^ Individuals considered high-risk, for example, those with immunosuppressive conditions or living with a vulnerable adult, were eligible earlier (adolescents in August 2021^3^ and children in January 2022^4^).

Authorization in children and adolescents was based on phase II/III randomized controlled trials showing high immunogenicity and efficacy against infection. However, protection against severe disease and safety endpoints were not assessed in randomized controlled trials.^5^ Multiple countries have reported rare cases of myocarditis and pericarditis following mRNA COVID-19 vaccines^6–8^: these events are listed in BNT162b2 product information.^9^

We used the OpenSAFELY-TPP database, covering 40% of English primary care practices and linked to national coronavirus surveillance, hospital episodes, and death registry data, to emulate a hypothetical target trial to evaluate the effectiveness of childhood COVID-19 vaccination against COVID-19-related and other outcomes.

## METHODS

### Data Source

We linked, stored, and analyzed all data securely using the OpenSAFELY platform, https://www.opensafely.org/, as part of the National Health Service England OpenSAFELY COVID-19 service. Data include pseudonymized data such as coded diagnoses, medications, and physiologic parameters. No free text data was included. All code is shared openly for review and re-use under the MIT open license (https://github.com/opensafely/vaccine-effectiveness-in-kids). Detailed pseudonymized patient data is potentially reidentifiable and therefore not shared. Primary care records managed by the general practitioner software provider TPP were linked to ONS death data through OpenSAFELY.

### Eligibility Criteria, Vaccination Groups, and Matching

We included (1) all adolescents aged 12–15 years and (2) all children aged 5–11 years on 31 August 2021, when age-based vaccine eligibility criteria were defined, who were not considered clinically vulnerable, as defined by the Joint Committee on Vaccination and Immunisation (clinically vulnerable individuals are those with certain chronic conditions—such as chronic respiratory, heart or neurological disease, or immunosuppressive conditions or medications—who were eligible for vaccination before the start of the study entry period); had been continuously registered at a general practitioner practice using TPP’s SystmOne clinical information system for 42 days; had no evidence of SARS-CoV-2 infection or COVID-19 disease within the 30 days before vaccination; and had complete information on sex, deprivation, ethnicity, and National Health Service region.

Using a matched sequential trials design, we estimated the effectiveness and safety of: (1) the first vaccine dose versus no vaccination and (2) a second dose versus a single dose only. For the first vaccine dose, on each day of the study period, we emulated a trial such that each eligible individual vaccinated with their first dose was matched 1:1 without replacement with an eligible individual who had not yet been vaccinated. If multiple matches were available, the match was selected at random. We matched unvaccinated individuals to at most one vaccinated individual, but were eligible for inclusion in the vaccination group later on if they were subsequently vaccinated. We excluded from the analysis unvaccinated people who were not matched at any point. Follow-up of the vaccinated group included the time after the second vaccination. We pooled vaccinated and unvaccinated groups from each trial, respectively, for analysis. We used the same approach to assess the effectiveness and safety of a second vaccine dose, among individuals who had received a first vaccine dose.

Matching criteria were: age within year on 31 August 2021 (i.e., in the same school-year), sex (male/female), region, deprivation (as defined by Index of Multiple Deprivation quintile), evidence of prior infection (yes/no), prior tests in the preceding 26 weeks (0, 1–2, 3+), prior non-COVID childhood vaccination (measles/mumps/rubella: yes/no), and all other childhood vaccines^10^ (yes/no) and, for the second dose comparison, first vaccination date within 7 days.

### Outcomes

We considered five effectiveness outcomes: positive SARS-CoV-2 test, COVID-19 accident and emergency (A&E) attendance, COVID-19 hospitalization, COVID-19 critical care admission, and COVID-19 death. Freely-available community testing for COVID-19 ended on 31 March 2022, and as non-high-risk children became eligible for vaccination in April 2022, the positive SARS-CoV-2 test was not considered for children (age 5–11 years). Non-COVID-19 death, fractures, and effectiveness in the first week after vaccination were considered as negative control outcomes (eBox 2; https://links.lww.com/EDE/C271).^11^ We also considered A&E attendance, unplanned hospitalization, pericarditis, and myocarditis as safety endpoints (eBox 1; https://links.lww.com/EDE/C271). Outcomes are described in eBoxes 1 and 2; https://links.lww.com/EDE/C271.

### Follow-up

Each individual was followed from assignment to a comparison group (“time zero”) until the earliest of: end of freely-available community testing (positive SARS-CoV-2 test only), outcome, death, practice deregistration, or 20 weeks. Additionally, both members of the matched pair were censored if the matched control was vaccinated (i.e., first dose vaccination of the unvaccinated control in the first dose analysis, or second dose vaccination of the single-dose-only control in the second dose analysis), to ensure follow-up is not systematically longer in the vaccinated group.

### Statistical Analysis

We estimated the cumulative incidence of events using the Kaplan–Meier estimator. We derived confidence intervals (CIs) using complementary log–log standard errors.

We also estimated period-specific incidence rates in each treatment group (number of events divided by person-time at risk), derived incidence rate ratios (IRRs), and their 95% CIs using Greenwood’s formula on the log-IRR scale. We also present vaccine effectiveness as a percentage, defined as 100 * (1 − IRR). We also derived 20-week risk differences and corresponding 95% CIs from the sum of squares of Greenwood standard errors.

We estimated effectiveness separately according to whether there was evidence of prior SARS-CoV-2 infection (eTables 1–4; https://links.lww.com/EDE/C271).

### Software, Code, and Reproducibility

Data management and analyses were conducted in Python version 3.8.10 (CreateSpace, Scotts Valley, CA) and R version 4.0.5 (R Foundation for Statistical Computing, Vienna, Austria). Code for data management and analysis, as well as codelists, is archived online https://github.com/opensafely/vaccine-effectiveness-in-kids.

### Disclosure Control

We redacted any reported figures based on counts below 8. Cumulative incidence plots are not reported for outcomes with fewer than 30 outcomes. To reduce reidentification risk, counts are reported to the nearest n * 6 − 3 (3, 9, 15, 21, …). All derived statistics are based on these rounded counts, including cumulative incidence curves, which are based on rounded numbers-at-risk and event counts.

## RESULTS

### Adolescents

Of 513,192 eligible adolescents who were registered at a TPP practice and received a BNT162b2 vaccination during the study period, 410,463 (80%) were matched with unvaccinated controls. (eFigure 1; https://links.lww.com/EDE/C271). Also, 220,929 (81%) of 271,440 eligible adolescents who received a second BNT162b2 vaccination were matched with single-vaccinated controls (eFigure 1; https://links.lww.com/EDE/C271).

As expected, the matching factors, other than age, were identically distributed in the vaccinated and control groups at the start of follow-up for all of the study populations (Table 1). Over 60% of adolescents in the first vaccination group received a second vaccination (eFigure 2; https://links.lww.com/EDE/C271). Most second vaccinations occurred at least 12 weeks after the first vaccination (eFigure 2; https://links.lww.com/EDE/C271).

**TABLE 1.** Characteristics n (%) of Matched Participants on the Day of Study Entry

	Adolescents				Children			
	First Dose vs. Unvaccinated		Second Dose vs. Single Dose Only		First Dose vs. Unvaccinated		Second Dose vs. Single Dose Only	
	First Dose (n = 410,463)	Unvaccinated (n = 410,463)	Second Dose (n = 220,929)	Single Dose Only (n = 220,929)	First Dose (n = 141,711)	Unvaccinated (n = 141,711)	Second Dose (n = 66,231)	Single Dose Only (n = 66,231)
Age								
5	-	-	-	-	3,537 (2.5)	2,457 (1.7)	237 (0.4)	153 (0.2)
6	-	-	-	-	13,887 (9.8)	13,905 (9.8)	5,859 (8.8)	5,811 (8.8)
7	-	-	-	-	15,837 (11.2)	15,591 (11.0)	6,909 (10.4)	6,843 (10.3)
8	-	-	-	-	17,865 (12.6)	18,435 (13.0)	8,181 (12.4)	8,187 (12.4)
9	-	-	-	-	22,497 (15.9)	21,759 (15.4)	10,329 (15.6)	10,293 (15.5)
10	-	-	-	-	26,505 (18.7)	26,187 (18.5)	12,639 (19.1)	12,633 (19.1)
11	-	-	-	-	37,311 (26.3)	31,473 (22.2)	15,855 (23.9)	15,783 (23.8)
12	79,083 (19.3)	78,669 (19.2)	28,353 (12.8)	25,401 (11.5)	4,275 (3.0)	11,901 (8.4)	6,219 (9.4)	6,531 (9.9)
13	103,227 (25.1)	104,391 (25.4)	54,291 (24.6)	55,527 (25.1)	-	-	3 (0.0)	0 (0.0)
14	103,773 (25.3)	103,527 (25.2)	56,595 (25.6)	56,829 (25.7)	-	-	-	-
15	104,895 (25.6)	105,753 (25.8)	56,685 (25.7)	59,901 (27.1)	-	-	-	-
16	19,485 (4.7)	18,123 (4.4)	24,999 (11.3)	23,265 (10.5)	-	-	-	-
Sex								
Female	205,173 (50.0)	205,173 (50.0)	110,121 (49.8)	110,121 (49.8)	70,611 (49.8)	70,611 (49.8)	33,159 (50.1)	33,159 (50.1)
Male	205,287 (50.0)	205,287 (50.0)	110,805 (50.2)	110,805 (50.2)	71,103 (50.2)	71,103 (50.2)	33,075 (49.9)	33,075 (49.9)
Deprivation								
1 (most deprived)	70,947 (17.3)	70,947 (17.3)	31,419 (14.2)	31,419 (14.2)	20,103 (14.2)	20,103 (14.2)	8,421 (12.7)	8,421 (12.7)
2	72,831 (17.7)	72,831 (17.7)	36,057 (16.3)	36,057 (16.3)	23,577 (16.6)	23,577 (16.6)	10,395 (15.7)	10,395 (15.7)
3	84,081 (20.5)	84,081 (20.5)	45,417 (20.6)	45,417 (20.6)	29,253 (20.6)	29,253 (20.6)	13,473 (20.3)	13,473 (20.3)
4	87,891 (21.4)	87,891 (21.4)	50,307 (22.8)	50,307 (22.8)	32,103 (22.7)	32,103 (22.7)	15,435 (23.3)	15,435 (23.3)
5 (least deprived)	94,707 (23.1)	94,707 (23.1)	57,723 (26.1)	57,723 (26.1)	36,675 (25.9)	36,675 (25.9)	18,513 (28.0)	18,513 (28.0)
Region								
East of England	99,471 (24.2)	99,471 (24.2)	55,581 (25.2)	55,581 (25.2)	35,019 (24.7)	35,019 (24.7)	17,493 (26.4)	17,493 (26.4)
London	18,519 (4.5)	18,519 (4.5)	8,697 (3.9)	8,697 (3.9)	6,147 (4.3)	6,147 (4.3)	2,271 (3.4)	2,271 (3.4)
Midlands	90,093 (21.9)	90,093 (21.9)	50,601 (22.9)	50,601 (22.9)	31,497 (22.2)	31,497 (22.2)	14,931 (22.5)	14,931 (22.5)
North East and Yorkshire	78,471 (19.1)	78,471 (19.1)	41,061 (18.6)	41,061 (18.6)	24,105 (17.0)	24,105 (17.0)	11,853 (17.9)	11,853 (17.9)
North West	36,057 (8.8)	36,057 (8.8)	18,333 (8.3)	18,333 (8.3)	10,005 (7.1)	10,005 (7.1)	3,981 (6.0)	3,981 (6.0)
South East	26,511 (6.5)	26,511 (6.5)	14,571 (6.6)	14,571 (6.6)	9,651 (6.8)	9,651 (6.8)	4,059 (6.1)	4,059 (6.1)
South West	61,335 (14.9)	61,335 (14.9)	32,079 (14.5)	32,079 (14.5)	25,275 (17.8)	25,275 (17.8)	11,637 (17.6)	11,637 (17.6)
Number of SARS-CoV-2 tests								
0	85,515 (20.8)	85,515 (20.8)	39,795 (18.0)	39,795 (18.0)	52,503 (37.0)	52,503 (37.0)	48,969 (73.9)	48,969 (73.9)
1–2	108,261 (26.4)	108,261 (26.4)	62,487 (28.3)	62,487 (28.3)	51,897 (36.6)	51,897 (36.6)	9,963 (15.0)	9,963 (15.0)
3+	216,681 (52.8)	216,681 (52.8)	118,647 (53.7)	118,647 (53.7)	37,311 (26.3)	37,311 (26.3)	7,299 (11.0)	7,299 (11.0)
Prior documented SARS-CoV-2 infection	82,137 (20.0)	82,137 (20.0)	50,007 (22.6)	50,007 (22.6)	65,307 (46.1)	65,307 (46.1)	30,777 (46.5)	30,777 (46.5)

Counts are based on values rounded up to the nearest n * 6 − 3, for disclosure control.

### Effectiveness of the First Dose in Adolescents

In 95,641 person-years of potential follow-up from 820,926 adolescents, there were 56,496 positive SARS-CoV-2 tests; 72 COVID-19 A&E attendances; 90 COVID-19 hospitalizations, of which three included admission to critical care; and no COVID-19 deaths. There were three non-COVID-19-related deaths; 3444 fractures; 22,764 A&E attendances; 2664 unplanned hospitalizations; nine cases of pericarditis; and three cases of myocarditis. All pericarditis and myocarditis events occurred in the first dose group, while all COVID-19-related critical care admissions were in the unvaccinated group. Further analyses were restricted to positive SARS-CoV-2 tests, COVID-19 A&E attendance, COVID-19 hospitalization, fractures, A&E attendance, and unplanned hospitalization.

**FIGURE 1.**
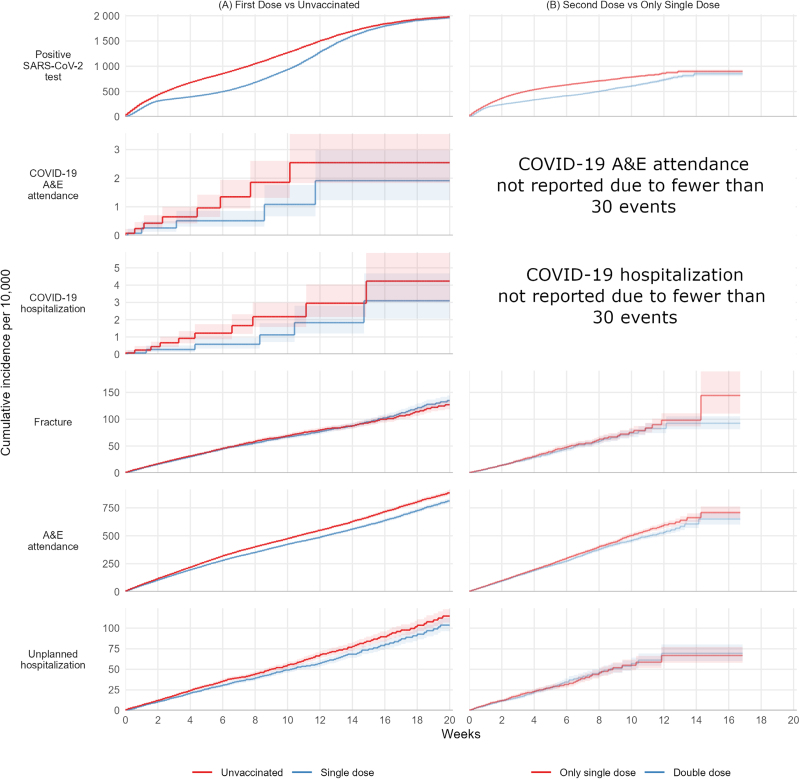
Kaplan–Meier estimates of cumulative incidence of outcomes in adolescents.

The rate of increase in the cumulative incidence of positive SARS-CoV-2 test after the first vaccine dose in adolescents declined substantially between 10 days and 6 weeks after vaccination, then increased (Figure 1). By 15 weeks, the cumulative incidence of positive SARS-CoV-2 tests was similar in the first-dose and unvaccinated groups. The 20-week risks per 10,000 were 1,961 (95% CI: 1,932, 1,990) and 1,979 (1,950, 2,008) in the vaccinated and unvaccinated groups, respectively. The IRR comparing vaccinated with unvaccinated adolescents was 0.74 (95% CI: 0.72, 0.75) and the vaccine effectiveness was 26.3 (25.1, 27.5) (Table 2).

**TABLE 2.** 20-Week Risks, Events per Person-Years, Risk Differences, and Incident Rate Ratios for Adolescents

Outcome	Events/Person-Years	20-Week Risk/10,000 (95% CI)	Events/Person-Years	20-Week Risk/10,000 (95% CI)	RD per 10,000 people (95% CI)	IRR (95% CI)	VE (95% CI)
First Dose vs. Unvaccinated (n = 410,463 per Group)							
	First Dose		Unvaccinated				
Positive SARS-CoV-2 test	25,287/45,064	1,961 (1,932-1,990)	31,209/40,990	1,979 (1,950, 2,008)	−18 (−59, 23)	0.74 (0.72, 0.75)	26.30 (25.10, 27.50)
COVID-19 A&E attendance	27/47,801	1.91 (1.23, 2.98)	45/47,827	2.54 (1.83, 3.54)	−0.63 (−1.83, 0.56)	0.60 (0.37, 0.97)	40.00 (3.30, 62.70)
COVID-19 hospitalization	33/47,799	3.09 (2.05, 4.67)	57/47,826	4.23 (3.05, 5.87)	−1.14 (−3.02, 0.74)	0.58 (0.38, 0.89)	42.10 (11.10, 62.30)
COVID-19 critical care	0/47,804	-	3/47,837	0.24 (0.08, 0.76)	−0.24 (−0.52, 0.03)	-	-
COVID-19 death	0/47,804	-	0/47,837	-	-	-	-
Non-COVID-19 death	3/47,804	0.51 (0.16, 1.58)	0/47,837	-	0.51 (−0.07, 1.09)	-	-
Fracture	1,713/47,624	135 (126, 144)	1,731/47,633	127 (119, 136)	8 (−4, 20)	0.99 (0.93, 1.06)	1.00 (−5.80, 7.40)
A&E attendance	10,743/46,601	812 (792, 834)	12,021/46,435	885 (864, 907)	−73 (−102, −43)	0.89 (0.87, 0.91)	10.90 (8.60, 13.20)
Unplanned hospitalization	31,245/47,671	104 (96, 112)	1,419/47,656	115 (107, 123)	−11 (−22, 0)	0.88 (0.81, 0.95)	12.30 (5.40, 18.70)
Pericarditis events	9/47,802	0.31 (0.16, 0.61)	0/47,837	-	0.31 (0.11, 0.52)	-	-
Myocarditis events	3/47,803	0.08 (0.03, 0.24)	0/47,837	-	0.08 (−0.01, 0.17)	-	-
Second Dose vs. Single Dose Only (n = 220,929 per Group)							
	Second Dose		Single Dose Only				
Positive SARS-CoV-2 test	6,243/14,788	850 (802, 899)	8,667/13,732	898 (861, 935)	−48 (−109, 13)	0.67 (0.65, 0.69)	33.10 (30.90, 35.30)
COVID-19 A&E attendance	3/15,220	0.14 (0.05, 0.44)	3/15,223	0.14 (0.04, 0.42)	0.01 (−0.22, 0.23)	1.00 (0.20, 4.96)	0.00 (−395.60, 79.80)
COVID-19 hospitalization	9/15,219	0.57 (0.29, 1.10)	15/15,222	2.02 (1.12, 3.62)	−1.45 (−2.69, −0.21)	0.60 (0.26, 1.37)	40.00 (−37.10, 73.70)
COVID-19 critical care	0/15,220	-	0/15,224	-	-	-	-
COVID-19 death	0/15,220	-	0/15,224	-	-	-	-
Non-COVID-19 death	0/15,220	-	0/15,224	-	-	-	-
Fracture	561/15,182	92 (81, 105)	597/15,184	144 (110, 189)	−52 (−92, −11)	0.94 (0.84, 1.05)	6.00 (−5.50, 16.20)
A&E attendance	3,615/14,982	651 (602, 703)	3,873/14,957	709 (658, 763)	−58 (−132, 15)	0.93 (0.89, 0.98)	6.80 (2.50, 10.90)
Unplanned hospitalization	435/15,194	69 (60, 80)	441/15,189	67 (58, 77)	2 (−11, 16)	0.99 (0.86, 1.13)	1.40 (−12.60, 13.60)
Pericarditis events	3/15,220	0.21 (0.07, 0.66)	0/15,224	-	0.21 (−0.03, 0.45)	-	-
Myocarditis events	0/15,220	-	0/15,224	-	-	-	-

Counts, risk, and survival estimates are based on values rounded up to the nearest n * 6 − 3, for disclosure control.

RD indicates risk difference; VE, vaccine effectiveness.

The incidence of COVID-19 A&E attendance was lower after the first vaccination than in the unvaccinated group (IRR: 0.60; 95% CI: 0.37, 0.97), and the vaccine effectiveness was 40.0 (3.3, 62.7). The 20-week risks per 10,000 were 1.91 (95% CI: 1.23, 2.98) and 2.54 (1.83, 3.54), respectively. The incidence of COVID-19 hospitalization was lower after the first vaccination than in the unvaccinated group (IRR: 0.58; 0.38, 0.89), and the vaccine effectiveness was 43.1 (11.1, 62.3). The 20-week risks per 10,000 were 3.09 (2.05, 4.67) and 4.23 (3.05, 5.87), respectively.

The incidence of fractures (negative control outcome) was similar in the first vaccination and unvaccinated groups (IRR: 0.99; 95% CI: 0.93, 1.06), and the vaccine effectiveness was 1.0 (−5.8, 7.4). The 20-week risks per 10,000 were 135 (95% CI: 126, 144) in the single-dose group and 127 (119, 136) in the unvaccinated group. The incidence of A&E attendance (safety outcome) was lower after the first vaccination than in the unvaccinated group (IRR: 0.89; 0.87, 0.91), and the vaccine effectiveness was 10.9 (8.6, 13.2). The 20-week risks per 10,000 were 812 (95% CI: 792, 834) in the single-dose group and 885 (864, 907) in the unvaccinated group. The incidence of unplanned hospitalization was lower after the first vaccination than in the unvaccinated group (IRR: 0.88; 0.81, 0.95), and the vaccine effectiveness was 12.3 (5.4, 18.7). The 20-week risks per 10,000 were 104 (96, 112) in the single-dose group and 115 (107, 123) in the unvaccinated group.

### Effectiveness of the Second Dose Versus the Single Dose Only in Adolescents

In 30,444 person-years follow-up from 441,858 adolescents after first vaccination, there were 14,910 positive SARS-CoV-2 tests; 6 COVID-19 A&E attendances; 24 COVID-19 hospitalizations, none of which included admission to critical care; and no COVID-19 deaths. There were no non-COVID-19-related deaths; 1158 fractures; 7488 A&E attendances; 876 unplanned hospitalizations; three cases of pericarditis; and no cases of myocarditis. All pericarditis events occurred in the single-dose group. Further analyses were restricted to positive SARS-CoV-2 tests, A&E attendance, fractures, and unplanned hospitalization.

The rate of increase in the cumulative incidence of positive SARS-CoV-2 test after the second dose declined between 10 days and 6 weeks after vaccination, then increased (Figure 1). By 14 weeks, the cumulative incidence of positive SARS-CoV-2 test was similar in the second and single-dose groups. The 20-week risks per 10,000 were 850 (95% CI: 802, 899) after the second dose and 898 (861, 935) after the single dose. The IRR comparing second with single doses was 0.67 (95% CI: 0.65, 0.69), and the vaccine effectiveness was 33.1 (30.9, 35.3) (Table 2).

The incidence of fractures (negative control outcome) was similar in the second and single-dose groups; the IRR was 0.94 (95% CI: 0.84, 1.05), and the vaccine effectiveness was 6.0 (5.5, 16.2). The 20-week risks per 10,000 were 92 (95% CI: 81, 105) in the second dose group and 144 (110, 189) in the single-dose group. The incidence of A&E attendance (safety outcome) was lower after the second than single dose (IRR: 0.93; 0.89, 0.98), and the vaccine effectiveness was 6.8 (2.5, 10.9). The 20-week risks per 10,000 were 651 (95% CI: 602, 703) in the second-dose group and 709 (658, 763) in the single-dose group. The incidence of unplanned hospitalization was similar in the second- and single-dose groups (IRR: 0.99; 0.86, 1.13), and the vaccine effectiveness was 1.4 (−12.6, 13.6). The 20-week risks per 10,000 were 69 (60, 80) in the second-dose group and 67 (58, 77) in the single-dose group (Table 2).

### Myocarditis and Pericarditis in Adolescents

Half or fewer (in order to comply with statistical disclosure control obligations and minimize the risk of reidentification of individuals, we do not report rates at higher precision) of adolescents diagnosed with pericarditis were admitted to hospital, and half or fewer attended A&E. More than half of adolescents with myocarditis were admitted to hospital, and more than half attended A&E. The maximum length of critical care admission was 1 day for either event. The maximum length of hospitalization stay was 0 days for pericarditis and 2 days for myocarditis. There were no deaths after these events.

### Children

Of 177,360 children eligible in the first vaccination group, 141,711 (80%) were matched with unvaccinated controls (eFigure 1; https://links.lww.com/EDE/C271). In addition, 66,231 (67%) of 99,102 children who received a second BNT162b2 vaccination were matched with single-vaccinated control (eFigure 1; https://links.lww.com/EDE/C271). Nearly 60% of children in the first vaccination group received a second vaccination (eFigure 2; https://links.lww.com/EDE/C271). Most second vaccinations occurred at least 12 weeks after the first vaccination (eFigure 2; https://links.lww.com/EDE/C271).

### Effectiveness of First Dose Versus Unvaccinated in Children

**FIGURE 2.**
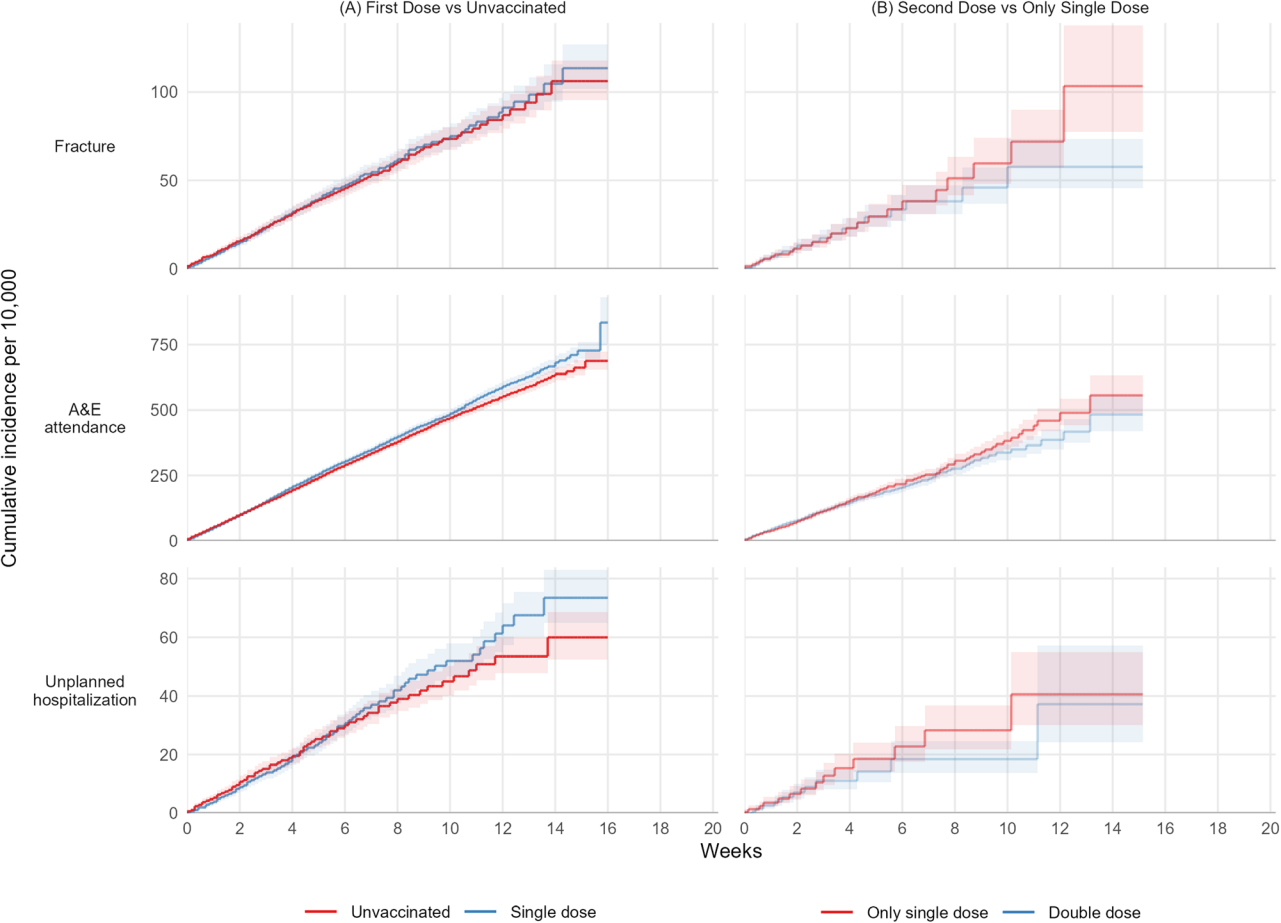
Kaplan–Meier estimates of cumulative incidence of outcomes in children.

In 32,476 person-years follow-up, there were no COVID-19 A&E attendances; six COVID-19 hospitalizations (none included admission to critical care), and no COVID-19 or non-COVID-19-related deaths. There were 1254 fractures; 8016 A&E attendances; 798 unplanned hospitalizations; three cases of pericarditis; and no cases of myocarditis. All pericarditis events occurred in the first dose group. Further analyses were restricted to fractures, A&E attendance, and unplanned hospitalization (Figure 2A).

The incidence of fractures was similar in the first vaccination and unvaccinated groups (IRR: 1.02; 95% CI: 0.91, 1.14), and the vaccine effectiveness was −2 (−13.9, 8.7). The 20-week risks per 10,000 were 113 (95% CI: 102, 127) in the single-dose group and 106 (95, 118) in the unvaccinated group. The incidence of A&E attendance was higher after the first vaccination than in the unvaccinated group (IRR: 1.05; 95% CI: 1.01, 1.10), and the vaccine effectiveness was −5.2 (−9.9, −0.7). The 20-week risks per 10,000 were 834 (748, 929) in the single-dose group and 687 (653, 723) in the unvaccinated group. The IRR comparing the incidence of unplanned hospitalization in the first vaccination and unvaccinated groups was 1.10 (95% CI: 0.95, 1.26), and the vaccine effectiveness was −9.5; −5.8, 4.7). The 20-week risks per 10,000 were 73 (65, 83) in the single-dose group and 60 (52, 69) in the unvaccinated group (Table 3).

**TABLE 3.** 20-Week Risks, Events per Person-Years, Risk Differences, and Incident Rate Ratios for Children

Outcome	Events/Person-Years	20-Week Risk/10,000 (95% CI)	Events/Person-Years	20-Week Risk/10,000 (95% CI)	RD per 10,000 People (95% CI)	IRR (95% CI)	VE (95% CI)
First Dose vs. Unvaccinated (n = 141,711 per Group)							
	First Dose		Unvaccinated				
COVID-19 A&E attendance	0/16,233	-	0/16,243	-	-	-	-
COVID-19 hospitalization	3/16,233	0.26 (0.08, 0.79)	3/16,242	0.27 (0.09, 0.85)	−0.02 (−0.44, 0.41)	1.00 (0.20, 4.96)	−0.10 (−395.70, 79.80)
COVID-19 critical care	0/16,233	-	0/16,243	-	-	-	-
COVID-19 death	0/16,233	-	0/16,243	-	-	-	-
Non-COVID-19 death	0/16,233	-	0/16,243	-	-	-	-
Fracture	633/16,175	113 (102, 127)	621/16,183	106 (95, 118)	7 (−9, 24)	1.02 (0.91, 1.14)	−2.00 (−13.90, 8.70)
A&E attendance	4,107/15,862	834 (738, 929)	3,909/15,878	687 (653, 723)	146 (49, 244)	1.05 (1.01, 1.10)	−5.20 (−9.90, −0.70)
Unplanned hospitalization	417/16,196	73 (65, 83)	381/16,204	60 (52, 69)	14 (1, 26)	1.10 (0.95, 1.26)	−9.50 (−25.80, 4.70)
Pericarditis events	3/16,233	0.22 (0.07, 0.67)	0/16,243	-	0.22 (−0.03, 0.46)	-	-
Myocarditis events	0/16,233	-	0/16,243	-	-	-	-
Second Dose vs. Single Dose Only (n = 66,231 per Group)							
	Second Dose		Single Dose Only				
COVID-19 A&E attendance	0/4,436	-	0/4,439	-	-	-	-
COVID-19 hospitalization	0/4,436	-	0/4,439	-	-	-	-
COVID-19 critical care	0/4,436	-	0/4,439	-	-	-	-
COVID-19 death	0/4,436	-	0/4,439	-	-	-	-
Non-COVID-19 death	0/4,436	-	0/4,439	-	-	-	-
Fracture	123/4,427	58 (45, 73)	141/4,431	103 (77, 138)	−46 (−78, −13)	0.87 (0.69, 1.11)	12.70 (−11.20, 31.40)
A&E attendance	819/4,387	483 (419, 557)	861/4,382	556 (488, 632)	−73 (−172, 27)	0.95 (0.86, 1.05)	5.00 (−4.50, 13.70)
Unplanned hospitalization	63/4,432	37 (24, 57)	81/4,431	41 (30, 55)	−3 (−24, 17)	0.78 (0.56, 1.08)	22.20 (−8.10, 44.10)
Pericarditis events	0/4,436	-	0/4,439	-	-	-	-
Myocarditis events	0/4,436	-	0/4,439	-	-	-	-

Counts and risk estimates are based on values rounded up to the nearest n * 6 − 3, for disclosure control.

RD indicates risk difference; VE, vaccine effectiveness.

### Effectiveness of Second Dose Versus Single Dose Only in Children

In 8875 person-years follow-up, there were no COVID-19 A&E attendances; no COVID-19 hospitalizations; no admissions to critical care; and no COVID-19 or non-COVID-19-related deaths. There were 264 fractures, 1680 A&E attendances, 144 unplanned hospitalizations, and no cases of pericarditis or myocarditis. Further analyses were restricted to fractures, A&E attendance, and unplanned hospitalization (Figure 2B).

The IRR comparing the incidence of fractures in the second- and single-dose groups was 0.87 (95% CI: 0.69, 1.11), and the vaccine effectiveness was 12.7 (−11.2, 31.4). The 20-week risks per 10,000 were 58 (95% CI: 45, 73) in the second-dose group and 103 (77, 138) in the single-dose group. The incidence of A&E attendance was similar in the second- and single-dose groups (IRR: 0.95; 0.86, 1.05), and the vaccine effectiveness was 5.0 (−4.5, 13.7). The 20-week risks per 10,000 were 483 (419, 557) and 556 (488, 632), respectively. The IRR comparing the incidence of unplanned hospitalization in the second- and single-dose groups was 0.78 (95% CI: 0.56, 1.08), and the vaccine effectiveness was 22.2 (−8.1, 44.1). The 20-week risks per 10,000 were 37 (24, 57) in the second-dose group and 41 (30, 55) in the single-dose group (Table 3).

### Myocarditis and Pericarditis Events in Children

No children experienced a myocarditis event; all three pericarditis events occurred after first vaccination and did not require hospitalization or critical care.

## DISCUSSION

This observational cohort study of COVID-19 vaccination with BNT162b2 in England was based on 410,463 adolescents (12–15 years old) receiving a first vaccination, 220,029 adolescents receiving a second vaccination, 141,711 children (5–11 years old) receiving a first vaccination, and 66,231 children receiving a second vaccination. We estimated an initial protective effect against a positive SARS-CoV-2 test in adolescents that waned by 14 weeks. The incidence of COVID-19 A&E attendance was lower after the first vaccination than in the unvaccinated group for adolescents, and COVID-19 A&E attendance was rare in the other adolescent group and the child groups. COVID-19-related hospitalization and critical care attendance were rare in both adolescents and children, and there were no COVID-19-related deaths. While rare, all myocarditis and pericarditis events during the study period occurred in vaccinated individuals: there were no deaths after myocarditis or pericarditis. The rate of fractures was similar across vaccine groups in both adolescents and children. None of the child cohort required hospitalization or critical care after a pericarditis event. In the adolescents, the maximum length of hospital admission was 1 day for critical care and 2 days for hospitalization.

Our findings provide insights into the balance between protection by vaccination against target outcomes (positive SARS-CoV-2 tests, COVID-19-related hospitalization, and A&E attendance) and the increased risk of pericarditis and myocarditis. In adolescents, the reduction in risk of COVID-19 hospitalization per 10,000 individuals (−1.14 for first dose vs. unvaccinated, −1.45 for second vs. first dose) was larger than the increase in risk of both myocarditis (0.08 for first dose vs. unvaccinated) and pericarditis (0.31 for first dose vs. unvaccinated, 0.21 for second vs. first dose). However, the reduction in risk of COVID-19 hospitalization in children (−0.02 for first dose vs. unvaccinated) was lower than the increase in risk of pericarditis (0.22).

### Strengths and Limitations

Our study has several limitations. First, positive SARS-CoV-2 test data underestimates the true incidence of infection. Both lateral flow tests and polymerase chain reaction tests were freely available in the United Kingdom until 31 March 2023, but many asymptomatic and symptomatic infections will not have been recorded.

Second, myocarditis and pericarditis following COVID-19 vaccination were publicly reported from May 2021.^12^ The vaccinations in our study were after this date, so there is a potential for ascertainment bias if diagnostic thresholds were lower in vaccinated than unvaccinated individuals.

Third, outcome ascertainment relies on routinely collected data rather than the rigorous, active case-finding often deployed in randomized trials. Consequently, potential differences in health-seeking behaviors, by children or their parents or carers, may mean that vaccinated children are more willing or able to present to healthcare services than unvaccinated children for mild symptoms or for SARS-CoV-2 testing. This could result in an underestimation of effectiveness and safety endpoints in the unvaccinated group compared with the vaccinated group, in turn underestimating vaccine effectiveness and vaccine safety for nonsevere endpoints.

Fourth, we excluded clinically vulnerable children and adolescents who were eligible for vaccination before the general roll-out, so our results may not be generalizable to this group. Vaccine safety and effectiveness in this group are important, but there are substantial challenges in controlling confounding arising from unmeasured severity of underlying conditions that could influence vaccine uptake and effectiveness. Sixth, due to small numbers, we did not study vaccine effectiveness in those who had received a vaccination other than BNT162b2.

Fifth, we did not look at safety events after SARS-CoV-2 infection, and so our study does not shed light on the relative safety of vaccination compared with infection.

Sixth, though the detailed data available in OpenSAFELY allowed us to match vaccinated individuals with control individuals on multiple characteristics, bias due to unmeasured confounding cannot be ruled out. For instance, differences in testing behavior between vaccinated and unvaccinated people may have contributed to the apparent waning effect if testing is more common among vaccinated individuals. Similarly, healthcare-seeking behaviors or access are likely to influence both vaccination and the desire or ability to seek care for mild to moderate symptoms such as those associated with myocarditis and pericarditis. Unmeasured differences in health-seeking behaviors may therefore account for some of the observed differences in safety endpoints. Further, it was not possible to reliably identify individuals with COVID-19 symptoms before testing or hospital admission, because this information is not routinely recorded in primary care records. Delayed vaccination in individuals with symptoms may explain the apparent effectiveness against positive tests during the first week (eFigures 3 and 4; https://links.lww.com/EDE/C271); however, the incidence of positive tests substantially reduced from 1 to 2 weeks in the vaccinated group (Figure 1A) compared with the unvaccinated group, consistent with a delayed but protective immunological response to vaccination. A similar pattern was observed for second-dose versus single-dose vaccination (Figure 1B). Lifestyle factors that may influence both health and vaccination are not fully recorded in health records. The slightly higher rate of fractures (negative control outcome) in single-vaccinated adolescents and children compared with the second dose group could indicate limited unmeasured confounding.

Finally, we made certain pragmatic statistical design decisions for computational expediency. The standard errors used to calculate CIs ignore the nonindependence between treatment groups due to the possibility of individuals experiencing outcomes in both unvaccinated and vaccinated groups. This is mitigated by including only those individuals who have no record of COVID-19 infection or disease within 30 days before the start of follow-up, but outcomes in the same individual across both groups are still possible. CIs may therefore be too narrow. We used pair-censoring to ensure balance in follow-up duration between treatment groups. However, informative censoring due to postbaseline factors may remain. Our use of matching leads to the exclusion of eligible participants. However, the generalizability of the results will only be affected if there are modifiers of vaccine effectiveness whose characteristics differ between vaccinated and unvaccinated participants.

### Findings in Context

BNT162b2 vaccination was shown to be effective in protecting against COVID-19 infection in a multinational phase 3 trial of 2260 adolescents aged 12–15 with a median follow-up of 2 months.^13,14^ Multiple observational studies have found that effectiveness wanes with time since vaccination.^15,16^ A recent systematic review found generally limited evidence regarding clinical outcomes of BNT162b2 vaccination among children and adolescents,^17^ although vaccination lowered hospitalization rates, including emergency admission.

Several studies have reported that children infected with SARS-CoV-2 appear to have the same degree of severity of illness as seen in adults.^18–20^ Most reported postvaccination cases of myocarditis and pericarditis in children and adolescents were mild.^21^ A self-controlled case-series study of 5.1 million children in England estimated 3 (95% CI: 0, 5) and 5 (95% CI: 3, 6) additional cases of myocarditis in adolescents per million following a first and second dose with BNT162b2, respectively.^22^ A systematic review found that myocarditis and pericarditis in children and adolescents were more prevalent among males and after the second dose of BNT162b2.^23^ While all cases of myocarditis and pericarditis in our study occurred in the vaccinated groups, we did not find higher rates of myocarditis or pericarditis after the second compared with the first dose. The rates of heart inflammation (myocarditis and pericarditis) in under 18s reported by the UK Medicines and Healthcare products Regulatory Agency were 13 and 8 per million after first and second doses, respectively, compared with our estimates of 27 and 10 per million, respectively. That there were no cases of myocarditis or pericarditis in the unvaccinated group does not mean that such events cannot occur without COVID-19 vaccination, only that these events were not observed in the unvaccinated groups in our specific matched analyses.

## CONCLUSIONS

This study found that initial protection by BNT162b2 vaccination against positive SARS-CoV-2 tests in adolescents aged 12–15 had waned by 14 weeks after vaccination. Rates of COVID-19 hospitalization and COVID-19 A&E attendance were lower after the first and second dose BNT162b2 vaccination in adolescents. Positive SARS-CoV-2 testing could not be considered for children. Severe outcomes were rare in children: there were fewer than seven (exact number redacted) COVID-19 hospitalizations and no COVID-19 A&E attendances, critical care admissions, or COVID-19 deaths.

## ACKNOWLEDGMENTS

We are very grateful for all the support received from the TPP Technical Operations team throughout this work, and for generous assistance from the information governance and database teams at NHS England and the NHS England Transformation Directorate.

## Supplementary Material



## References

[1] NHS. NHS Rolls Out COVID-19 Jab to Children Aged 12 to 15. NHS England. Available at: https://www.england.nhs.uk/2021/09/nhs-rolls-out-COVID-19-jab-to-children-aged-12-to-15/. Accessed 3 August 2025.

[2] NHS Rolls Out COVID Vaccine to Five Million 5 to 11 Year Olds. NHS England. Available at: https://www.england.nhs.uk/2022/04/nhs-rolls-out-COVID-vaccine-to-five-million-5-to-11-year-olds/. Accessed 3 August 2025.

[3] NHS to Rollout Boosters to Most at Risk 12 to 15 Year-Olds. NHS England. Available at: https://www.england.nhs.uk/2022/01/nhs-to-rollout-boosters-to-most-at-risk-12-to-15-year-olds/. Accessed 3 August 2025.

[4] NHS. NHS Expands COVID Vaccinations to the Most Vulnerable 5 to 11-Year-Olds. Available at: https://www.england.nhs.uk/2022/01/nhs-expands-COVID-vaccinations-to-the-most-vulnerable-5-to-11-year-olds/. Accessed 3 August 2025.

[5] Walter EB Talaat KR Sabharwal C. Evaluation of the BNT162b2 COVID-19 vaccine in children 5 to 11 years of age. N Engl J Med. 2022;386:3546. 10.1056/NEJMoa2116298 PMC8609605 34752019

[6] COVID-19 VaST Technical Report May 24, 2021. CDC; 2022 May. Available at: https://archive.cdc.gov/#/details?url=https://www.cdc.gov/vaccines/acip/work-groups-vast/report-2021-05-24.html. Accessed 3 September 2025.

[7] Mevorach D Anis E Cedar N. Myocarditis after BNT162b2 vaccination in Israeli adolescents. N Engl J Med. 2022;386:998–999. 35081295 10.1056/NEJMc2116999 PMC8823652

[8] Husby A Hansen JV Fosbøl E. SARS-CoV-2 vaccination and myocarditis or myopericarditis: population based cohort study. BMJ. 2021;375:e068665. 34916207 10.1136/bmj-2021-068665 PMC8683843

[9] Vaccine PNC. Fact Sheet for Health Workers. Available at: https://apps.who.int/iris/bitstream/handle/10665/343082/WHO-EURO-2021-1964-41715-59312-eng.pdf?sequence=1. Accessed 3 August 2025.

[10] Bennett Institute for Applied Data Science. *VACCINATION* Codelist Development: Childhood vaccination. Available at: https://github.com/opensafely/codelist-development/issues/254. Accessed 3 August 2025.

[11] Lipsitch M Tchetgen Tchetgen E Cohen T. Negative controls: a tool for detecting confounding and bias in observational studies. Epidemiology. 2010;21:383–388. 20335814 10.1097/EDE.0b013e3181d61eeb PMC3053408

[12] Marshall M Ferguson ID Lewis P. Symptomatic acute myocarditis in 7 adolescents after Pfizer-BioNTech COVID-19 vaccination. Pediatrics. 2021;148:e2021052478. 34088762 10.1542/peds.2021-052478

[13] Frenck RWJ Klein NP Kitchin N. Safety, immunogenicity, and efficacy of the BNT162b2 COVID-19 vaccine in adolescents. N Engl J Med. 2021;385:239250. 10.1056/NEJMoa2107456 PMC8174030 34043894

[14] Thomas SJ Moreira EDJ Kitchin N. Six month safety and efficacy of the BNT162b2 mRNA COVID-19 vaccine. 2021.

[15] Wu Q Tong J Zhang B. Real-world effectiveness of BNT162b2 against infection and severe diseases in children and adolescents. Ann Intern Med. 2024;177:165. 38190711 10.7326/M23-1754 PMC11956830

[16] Powell AA Kirsebom F Stowe J. Protection against symptomatic infection with delta (B.1.617.2) and omicron (B.1.1.529) BA.1 and BA.2 SARS-CoV-2 variants after previous infection and vaccination in adolescents in England, August, 2021-March, 2022: a national, observational, test-negative, case-control study. Lancet Infect Dis. 2023;23:435–444. 36436536 10.1016/S1473-3099(22)00729-0 PMC10032664

[17] Sabu JM Zahid I Jacob N Alele FO Malau-Aduli BS. Effectiveness of the BNT162b2 (Pfizer-BioNTech) vaccine in children and adolescents: a systematic review and meta-analysis. Vaccines (Basel). 2022;10:1880. 36366387 10.3390/vaccines10111880 PMC9698079

[18] Feldstein LR Tenforde MW Friedman KG; Overcoming COVID-19 Investigators. Characteristics and outcomes of US children and adolescents with multisystem inflammatory syndrome in children (MIS-C) compared with severe acute COVID-19. JAMA. 2021;325:1074. 33625505 10.1001/jama.2021.2091 PMC7905703

[19] Wu Z McGoogan JM. Characteristics of and important lessons from the Coronavirus Disease 2019 (COVID-19) outbreak in China: summary of a report of 72 314 cases from the Chinese Center for Disease Control and Prevention. JAMA. 2020;323:1239. 32091533 10.1001/jama.2020.2648

[20] Dawood FS Porucznik CA Veguilla V. Incidence rates, household infection risk, and clinical characteristics of SARS-CoV-2 infection among children and adults in Utah and New York City, New York. JAMA Pediatr. 2022;176:59. 34623377 10.1001/jamapediatrics.2021.4217 PMC8501415

[21] Das BB Moskowitz WB Taylor MB Palmer A. Myocarditis and pericarditis following mRNA COVID-19 vaccination: what do we know so far? Children. 2021;8:607. 34356586 10.3390/children8070607 PMC8305058

[22] Copland E Patone M Saatci D. Safety outcomes following COVID-19 vaccination and infection in 5.1 million children in England. Nat Commun. 2024;15:3822. 38802362 10.1038/s41467-024-47745-z PMC11130197

[23] Fatima M Khan MHA Ali MS. Development of myocarditis and pericarditis after COVID-19 vaccination in children and adolescents: a systematic review. Clin Cardiol. 2023;46:243–259. 36594165 10.1002/clc.23965 PMC10018089

