# Supplementary material for: OpenSAFELY: Effectiveness of COVID-19 Vaccination in Children and Adolescents
Source: Epidemiology. 2025 Sep 23;37(1):141–51. doi: 10.1097/EDE.0000000000001908 (PMC12643559; doi:10.1097/EDE.0000000000001908)
Supplement: Supplementary file 1 [file ede-37-141-s001.pdf]

Supplementary material:

Supplementary Table 1: Adolescents First Dose vs Unvaccinated subgroup analyses according to prior infection/COVID

First Dose vs Unvaccinated								
		N per	Unvaccinated		First Dose		RD per 10,000 people	
Outcome	Sub-group	group	Events / Person-years	20-week risk / 10,000 (95% CI)	Events / Person-years	20-week risk / 10,000 people (95% CI)	(95% CI)	IRR (95% CI)
Positive SARS-CoV-2 test	No prior SARS-CoV-2 infection	328,323	29,223 / 31,024	2,255 (2,221 to 2,289)	23,943 / 34,851	2,263 (2,221 to 2,289)	8 (−39 to 56)	0.73 (0.72 to 0.74)
	Prior SARS-CoV-2 infection	82,137	1,983 / 9,965	1,029 (974 to 1,087)	1,341 / 10,212	717 (974 to 1,087)	−313 (−385 to −240)	0.66 (0.62 to 0.71)
COVID-19 A&E attendance	No prior SARS-CoV-2 infection	328,323	45 / 37,486	4.16 (2.86 to 6.03)	21 / 37,466	1.54 (0.95 to 2.51)	−2.61 (−4.33 to −0.89)	0.47 (0.28 to 0.78)
	Prior SARS-CoV-2 infection	82,137	3 / 10,340	0.76 (0.24 to 2.35)	3 / 10,334	0.47 (0.15 to 1.45)	−0.29 (−1.30 to 0.72)	1.00 (0.20 to 4.96)
COVID-19 hospitalisation	No prior SARS-CoV-2 infection	328,323	51 / 37,484	4.04 (2.93 to 5.57)	33 / 37,465	5.46 (3.44 to 8.66)	1.42 (−1.41 to 4.25)	0.65 (0.42 to 1.00)
	Prior SARS-CoV-2 infection	82,137	3 / 10,340	0.53 (0.17 to 1.63)	3 / 10,334	0.54 (0.18 to 1.68)	0.02 (−0.84 to 0.87)	1.00 (0.20 to 4.96)
Fracture	No prior SARS-CoV-2 infection	328,323	1,335 / 37,334	124 (115 to 133)	1,311 / 37,328	131 (115 to 133)	7 (−6 to 20)	0.98 (0.91 to 1.06)
	Prior SARS-CoV-2 infection	82,137	399 / 10,298	150 (128 to 175)	399 / 10,296	149 (128 to 175)	−1 (−33 to 30)	1.00 (0.87 to 1.15)
A&E attendance	No prior SARS-CoV-2 infection	328,323	9,219 / 36,404	864 (841 to 887)	8,097 / 36,553	780 (841 to 887)	−83 (−116 to −51)	0.87 (0.85 to 0.90)
	Prior SARS-CoV-2 infection	82,137	2,799 / 10,030	977 (925 to 1,032)	2,643 / 10,047	945 (925 to 1,032)	−33 (−108 to 42)	0.94 (0.89 to 0.99)
Unplanned hospitalisation	No prior SARS-CoV-2 infection	328,323	1,095 / 37,355	114 (105 to 123)	939 / 37,367	100 (105 to 123)	−14 (−27 to −2)	0.86 (0.79 to 0.94)
	Prior SARS-CoV-2 infection	82,137	327 / 10,300	126 (107 to 148)	309 / 10,303	130 (107 to 148)	4 (−25 to 33)	0.94 (0.81 to 1.10)

20-week risks, events per person years, risk differences and incident rate ratios for adolescents, first vaccination). Counts and risk estimates are based on values rounded up to the nearest n*6-3, for disclosure control. IRR Incidence Rate Ratio, RD = Risk Difference, (95% Confidence Intervals). Counts and risk estimates are based on values rounded up to the nearest n*6-3, for disclosure control.

Supplementary Table 2: Adolescents Second Dose vs Single Dose Only subgroup analyses according to prior infection/COVID

Second Dose vs Single Dose Only								
			Single Dose Only		Second Dose			
Outcome	Sub-group	N per group	Events / Person-years	20-week risk / 10,000 people (95% CI)	Events / Person-years	20-week risk / 10,000 people (95% CI)	RD per 10,000 people (95% CI)	IRR (95% CI)
Positive SARS-CoV-2 test	No prior SARS-CoV-2 infection	170,919	7,827 / 10,317	1,061 (1,012 to 1,112)	5,661 / 11,286	1,144 (1,012 to 1,112)	83 (−88 to 253)	0.66 (0.64 to 0.68)
	Prior SARS-CoV-2 infection	50,007	843 / 3,414	449 (395 to 510)	585 / 3,501	341 (395 to 510)	−108 (−184 to −31)	0.68 (0.61 to 0.75)
COVID-19 A&E attendance	No prior SARS-CoV-2 infection	170,919	3 / 11,687	0.18 (0.06 to 0.54)	3 / 11,684	0.19 (0.06 to 0.57)	0.01 (−0.28 to 0.30)	1.00 (0.20 to 4.96)
	Prior SARS-CoV-2 infection	50,007	0 / 3,536	-	0 / 3,535	-	-	-
COVID-19 hospitalisation	No prior SARS-CoV-2 infection	170,919	9 / 11,686	0.95 (0.48 to 1.87)	9 / 11,684	0.83 (0.42 to 1.62)	−0.12 (−0.97 to 0.73)	1.00 (0.40 to 2.52)
	Prior SARS-CoV-2 infection	50,007	3 / 3,535	0.60 (0.19 to 1.86)	3 / 3,535	0.70 (0.23 to 2.18)	0.10 (−0.94 to 1.15)	1.00 (0.20 to 4.96)
Fracture	No prior SARS-CoV-2 infection	170,919	435 / 11,657	121 (98 to 147)	417 / 11,655	97 (98 to 147)	−23 (−52 to 6)	0.96 (0.84 to 1.10)
	Prior SARS-CoV-2 infection	50,007	159 / 3,526	81 (66 to 101)	147 / 3,526	105 (66 to 101)	23 (−11 to 58)	0.92 (0.74 to 1.16)
A&E attendance	No prior SARS-CoV-2 infection	170,919	2,793 / 11,490	685 (634 to 740)	2,619 / 11,506	654 (634 to 740)	−31 (−122 to 61)	0.94 (0.89 to 0.99)
	Prior SARS-CoV-2 infection	50,007	1,077 / 3,466	679 (609 to 758)	999 / 3,476	1,007 (609 to 758)	327 (71 to 584)	0.93 (0.85 to 1.01)
Unplanned hospitalisation	No prior SARS-CoV-2 infection	170,919	315 / 11,662	59 (51 to 68)	315 / 11,665	66 (51 to 68)	7 (−6 to 20)	1.00 (0.86 to 1.17)
	Prior SARS-CoV-2 infection	50,007	123 / 3,526	66 (52 to 84)	117 / 3,528	61 (52 to 84)	−5 (−26 to 15)	0.95 (0.74 to 1.22)

Table 2: 20-week risks, events per person years, risk differences and incident rate ratios for adolescents, second vaccination. Counts and risk estimates are based on values rounded up to the nearest n*6-3, for disclosure control. IRR Incidence Rate Ratio, RD = Risk Difference, (95% Confidence Intervals). Counts and risk estimates are based on values rounded up to the nearest n*6-3, for disclosure control.

Supplementary Table 3: Children First Dose vs Unvaccinated subgroup analyses according to prior infection/COVID

First Dose vs Unvaccinated								
			Unvaccinated		First Dose			
Outcome	Sub-group	N per group	Events / Person-years	20-week risk per 10,000 people (95% CI)	Events / Person-years	20-week risk per 10,000 people (95% CI)	RD per 10,000 people (95% CI)	IRR (95% CI)
COVID-19 hospitalisation	No prior SARS-CoV-2 infection	76,407	3 / 8,759	0.69 (0.22 to 2.14)	3 / 8,751	0.48 (0.15 to 1.49)	−0.21 (−1.16 to 0.74)	1.00 (0.20 to 4.96)
	Prior SARS-CoV-2 infection	65,307	3 / 7,485	0.59 (0.19 to 1.82)	0 / 7,482	-	−0.59 (−1.25 to 0.08)	-
Fracture	No prior SARS-CoV-2 infection	76,407	309 / 8,728	96 (83 to 112)	309 / 8,723	104 (83 to 112)	7 (−15 to 30)	1.00 (0.85 to 1.17)
	Prior SARS-CoV-2 infection	65,307	315 / 7,457	137 (113 to 166)	327 / 7,453	139 (113 to 166)	2 (−34 to 38)	1.04 (0.89 to 1.21)
A&E attendance	No prior SARS-CoV-2 infection	76,407	1,959 / 8,576	658 (610 to 710)	2,061 / 8,562	1,094 (610 to 710)	436 (112 to 759)	1.05 (0.99 to 1.12)
	Prior SARS-CoV-2 infection	65,307	1,947 / 7,303	704 (661 to 750)	2,049 / 7,301	850 (661 to 750)	146 (52 to 240)	1.05 (0.99 to 1.12)
Unplanned hospitalisation	No prior SARS-CoV-2 infection	76,407	201 / 8,738	55 (47 to 64)	207 / 8,732	63 (47 to 64)	8 (−5 to 20)	1.03 (0.85 to 1.25)
	Prior SARS-CoV-2 infection	65,307	177 / 7,467	55 (46 to 66)	207 / 7,465	77 (46 to 66)	22 (6 to 38)	1.17 (0.96 to 1.43)
Pericarditis events	No prior SARS-CoV-2 infection	76,407	0 / 8,759	-	0 / 8,752	-	-	-
	Prior SARS-CoV-2 infection	65,307	0 / 7,485	-	3 / 7,482	0.47 (0.15 to 1.46)	0.47 (−0.06 to 1.00)	-

20-week risks, events per person years, risk differences and incident rate ratios for children first vaccination. Counts and risk estimates are based on values rounded up to the nearest n*6-3, for disclosure control. IRR Incidence Rate Ratio, RD = Risk Difference, (95% Confidence Intervals). Counts and risk estimates are based on values rounded up to the nearest n*6-3, for disclosure control.

Supplementary Table 4: Children Second Dose vs Single Dose Only subgroup analyses according to prior infection/COVID

Second Dose vs Single Dose Only								
			Single Dose Only		Second Dose			
Outcome		N per group	Events / Person-years	20-week risk per 10,000 people (95% CI)	Events / Person-years	20-week risk per 10,000 people (95% CI)	RD per 10,000 people (95% CI)	IRR (95% CI)
Fracture	No prior SARS-CoV-2 infection	35,451	63 / 2,431	73 (53 to 103)	57 / 2,428	43 (53 to 103)	−31 (−59 to −3)	0.91 (0.63 to 1.30)
	Prior SARS-CoV-2 infection	30,777	75 / 1,999	84 (63 to 113)	63 / 1,998	59 (63 to 113)	−25 (−56 to 6)	0.84 (0.60 to 1.17)
A&E attendance	No prior SARS-CoV-2 infection	35,451	447 / 2,405	554 (459 to 669)	423 / 2,407	531 (459 to 669)	−24 (−175 to 128)	0.95 (0.83 to 1.08)
	Prior SARS-CoV-2 infection	30,777	417 / 1,976	969 (666 to 1,400)	399 / 1,980	547 (666 to 1,400)	−422 (−808 to −36)	0.96 (0.83 to 1.10)
Unplanned hospitalisation	No prior SARS-CoV-2 infection	35,451	45 / 2,431	82 (48 to 140)	27 / 2,431	15 (48 to 140)	−67 (−111 to −23)	0.60 (0.37 to 0.97)
	Prior SARS-CoV-2 infection	30,777	39 / 1,999	37 (25 to 55)	33 / 2,001	63 (25 to 55)	26 (−14 to 65)	0.85 (0.53 to 1.34)

20-week event counts, Kaplan-Meier risk estimates, and comparisons (children, first vaccination). Counts and risk estimates are based on values rounded up to the nearest n*6-3, for disclosure control. IRR Incidence Rate Ratio, RD = Risk Difference, (95% Confidence Intervals). Counts and risk estimates are based on values rounded up to the nearest n*6-3, for disclosure control.

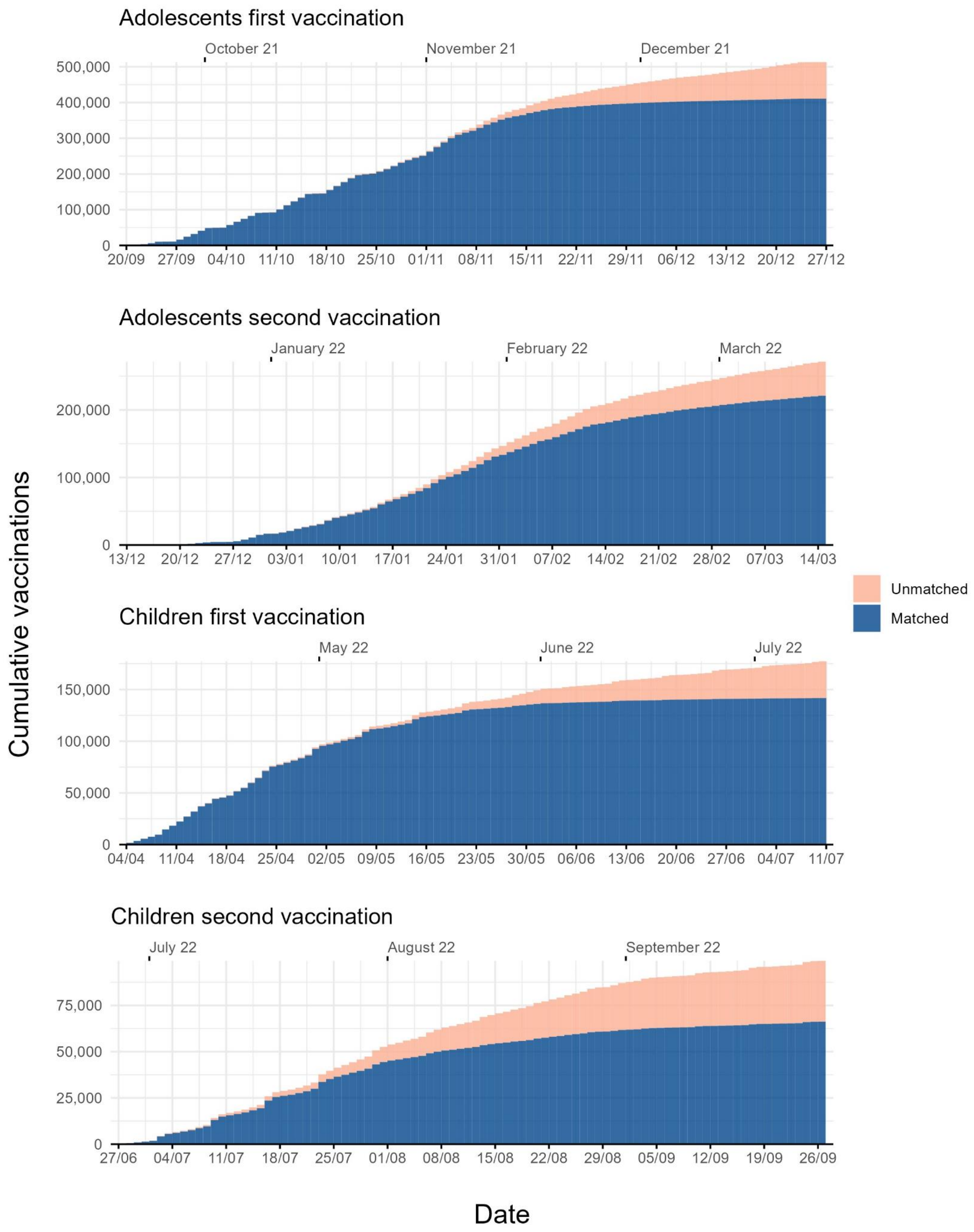

Supplementary Figure 1: Cumulative matching coverage

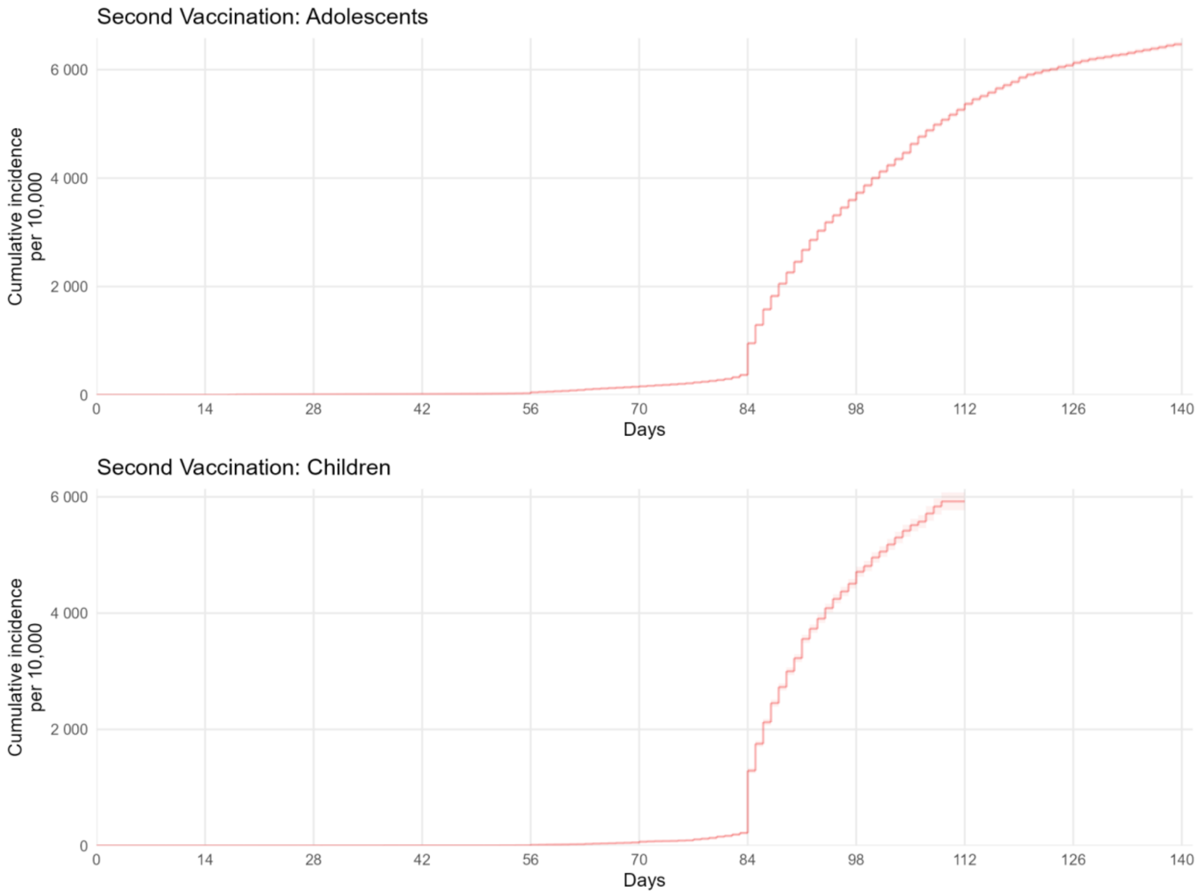

Supplementary Figure 2: Kaplan-Meier estimates of cumulative incidence of second vaccination for adolescents and children

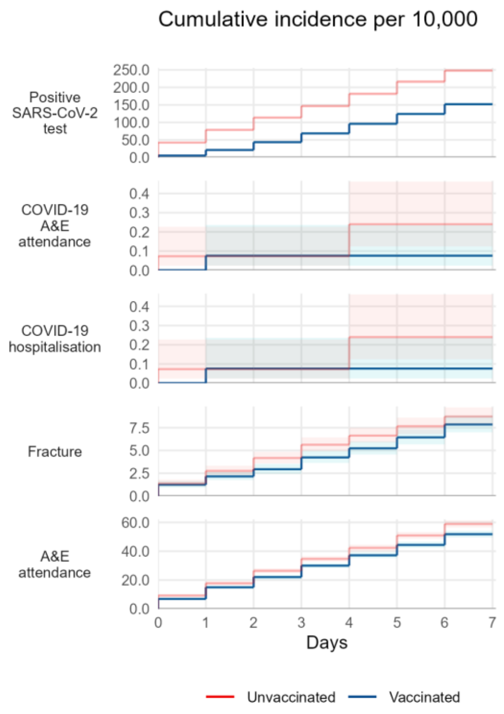

Supplementary Figure 3: Early outcome Kaplan-Meier estimates of cumulative incidence of outcomes in matched vaccinated and unvaccinated adolescents for the first 7 days.

(2)

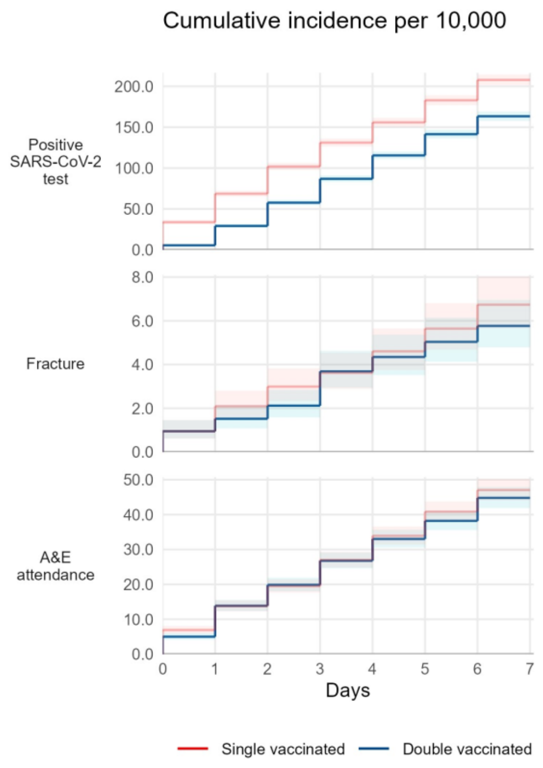

Supplementary Figure 4: Early outcome Kaplan-Meier estimates of cumulative incidence of outcomes in matched second vaccinated and single vaccinated adolescents for the first 7 days.

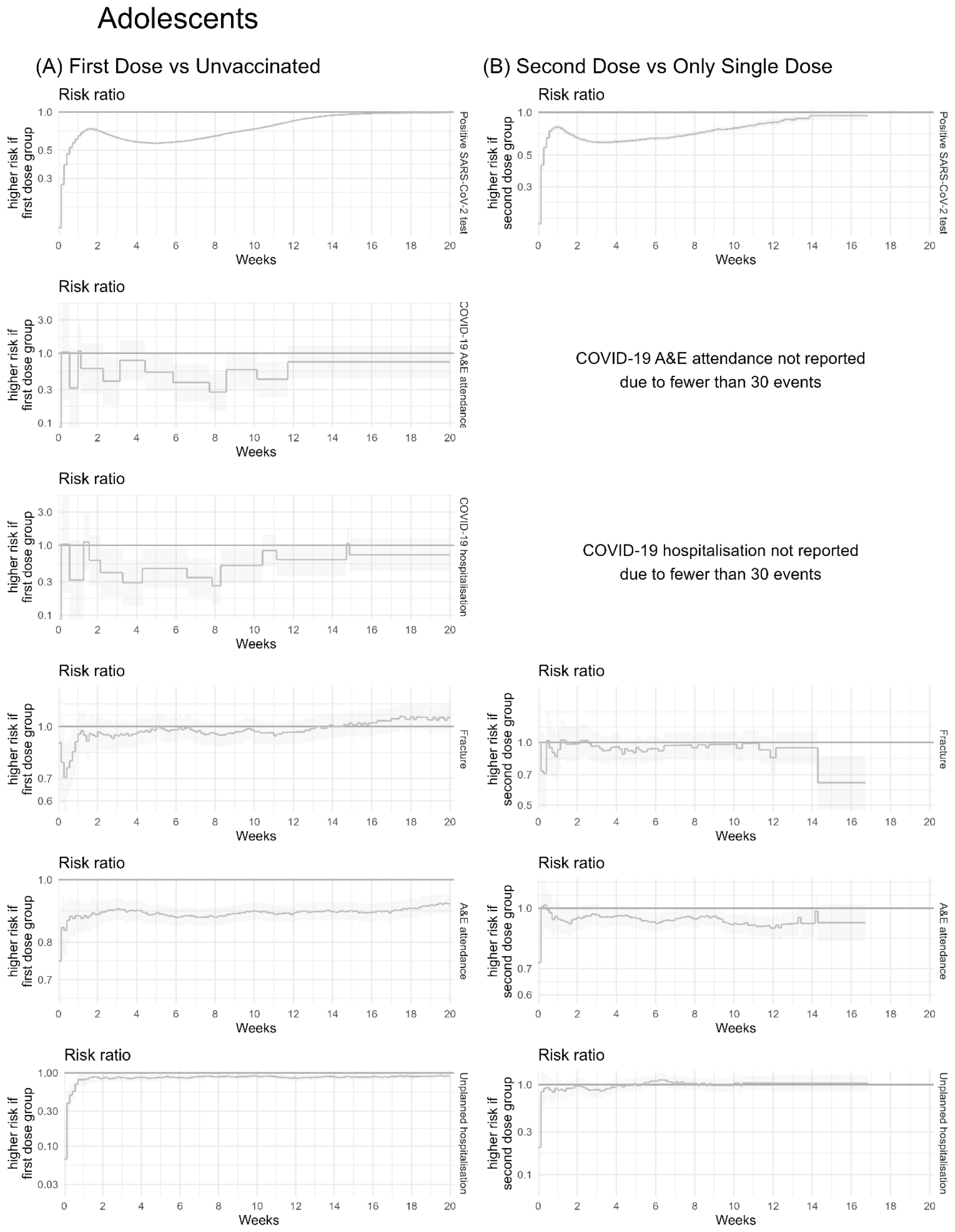

Supplementary Figure 5: Incident Rate Ratios of outcomes

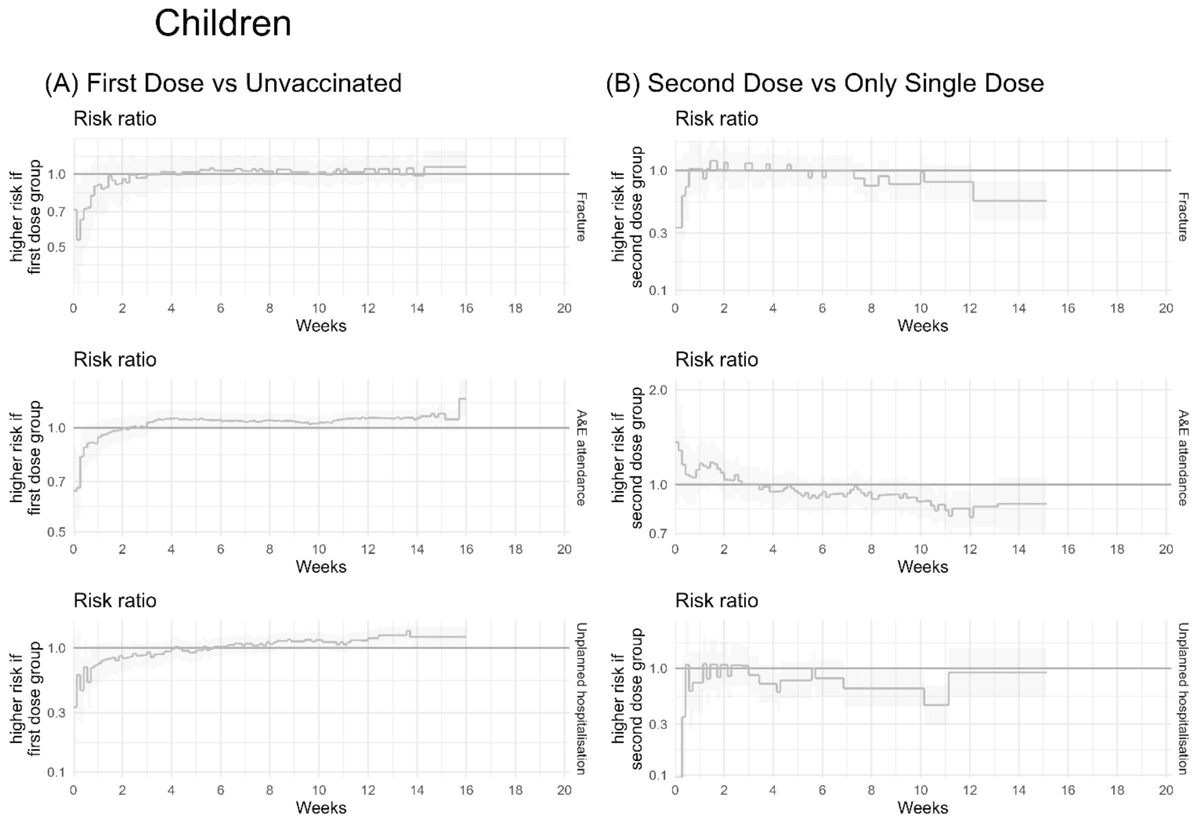

Supplementary Figure 6: Incident Rate Ratios of outcomes

Outcome	Definition
Positive SARS-CoV-2 test (Adolescents only)	Identified using SGSS testing records and based on swab date. Both polymerase chain reaction (PCR) and lateral flow test results are included, without differentiation between symptomatic and asymptomatic infection.
COVID-19 A&E attendances	Identified using HES emergency care records with U07.1 (“COVID-19, virus identified”) or U07.2 (“COVID-19, virus not identified”) ICD-10 diagnosis codes.
COVID-19 related hospital admission,	Identified using HES in-patient hospital records with U07.1 or U07.2 ICD-10 reason for admission codes.
COVID-19 critical care admission.	Hospital admissions involving a stay of at least one day on an ICU/HDU/critical care ward.
COVID-19 death,	Identified using death records with U07.1 or U07.2 ICD-10 codes present (i.e., as an underlying or contributing cause of death).
Non-COVID-19 death	negative control outcomes(Lipsitch et al., 2010)
Fracture	negative control outcomes(Lipsitch et al., 2010)
A&E attendances	Any recorded attendance of A&E from the Emergency Care Data Set (ECDS) during the study period.
Unplanned hospitalisation	Identified using HES in-patient hospital records
Pericarditis	Identified as either A&E attendances identified using HES emergency care records with 3238004 (“Pericarditis”) or 373945007 (“Pericardial effusion”) SNOMED diagnosis codes or hospital admission, identified using HES in-patient hospital records with I30 (“Acute pericarditis”) ICD-10 reason for admission codes.
Myocarditis	Identified as either A&E attendances identified using HES emergency care records with 50920009 (“myocarditis”) SNOMED diagnosis codes or hospital admission, identified using HES in-patient hospital records with I514 (“myocarditis, unspecified”), I41 (“myocarditis in diseases classified elsewhere”), or I40 (“Acute myocarditis”) ICD-10 reason for admission codes.

Supplementary Box 1: Definitions of study outcomes

Negative Control outcome

The protective effects of COVID-19 vaccination, if real, should be limited to outcomes plausibly linked to SARS-CoV-2 infection and COVID-19 disease. In contrast, if the relationship were due to uncontrolled confounding, then the same effect might be observed for irrelevant outcomes. To investigate the possibility of inadequately controlled confounding we also investigated the fractures and non-COVID-19 related death as a negative control outcomes:

- Fracture, identified using either death records with codes from fractures ICD-10 codelist present; hospital admission identified using HES in-patient hospital records with fractures ICD-10 codelist reason present for admission codes; or A&E attendances identified using HES emergency care records with fractures SNOMED codelist diagnosis codes.
- Non-COVID-19 death, identified using death records with U07.1 or U07.2 ICD-10 codes absent.

Similarly, we would not expect any effects of vaccination in the first week due to delayed immunological response to vaccination, and so we consider effectiveness during this period as an additional negative control outcome.

Supplementary Box 2: Definitions of Negative control outcomes
